# Cerebral activity manipulation of low-frequency repetitive transcranial magnetic stimulation in post-stroke patients with cognitive impairment

**DOI:** 10.3389/fneur.2022.951209

**Published:** 2022-11-08

**Authors:** Bi Yingli, Gong Zunke, Chen Wei, Wang Shiyan

**Affiliations:** ^1^Xuzhou Rehabilitation Hospital, Xuzhou, China; ^2^Department of Rehabilitation Medicine, Xuzhou Central Hospital, Xuzhou, China

**Keywords:** stroke, cognitive impairment, repetitive transcranial magnetic stimulation (rTMS), LOTCA, P300

## Abstract

**Objective:**

The aim of this study was to evaluate the therapeutic effect of low-frequency repetitive transcranial magnetic stimulation (rTMS) on post-stroke cognitive impairment (PSCI).

**Methods:**

Thirty-six PSCI patients were randomly divided into treatment and control groups of equal size. Both groups were pre-treated with conventional cognitive rehabilitation training. Subsequently, the treatment group was exposed to 1 Hz low-frequency repetitive transcranial magnetic stimulations for 8 weeks, with 5 days per week. Meanwhile, the control group was treated with placebo stimulations. Patients were evaluated *via* the LOTCA scale assessments and changes in P300 latencies and amplitudes before and after 8 weeks of treatment.

**Results:**

Before treatment, there were no significant differences between the two groups in LOTCA scores, P300 latencies, and amplitudes (*P* > 0.05). After treatment, LOTCA scores for both groups improved (*P* < 0.05), and those of the treatment group were higher than those of the control (*P* < 0.05). For both groups, P300 latencies were not only shortened but also had greater amplitudes (*P* < 0.05), and those for the treatment group were significantly shorter and larger than those of the control (*P* < 0.05).

**Conclusion:**

As a therapy, rTMS improved cognitive function in PSCI patients, possibly *via* regulation of neural electrical activity of the cerebral cortex.

## Introduction

Post-stroke cognitive impairment (PSCI) refers to a series of syndromes in which impairment of one cognitive area of executive function, attention, memory, language ability, and visual-spatial ability occurs within 6 months after a stroke (1). PSCI has a high incidence that can be as high as 80% (2) and greatly reduces a patient's quality of life and social participation (3), imposing a great burden on their families and society.

At present, research focus on the treatment of PSCI is increasing because active and effective treatment can improve cognitive function. Besides drug therapy, the other conventional treatment method involves non-drug therapy such as cognitive function training, but the effects are limited. A new research-based intervention is repetitive transcranial magnetic stimulation (rTMS); it is gradually being applied in clinics, which can directly stimulate the cerebral cortex to treat dyskinesia (4), aphasia, dysphagia (5, 6), and other dysfunctions after a stroke. As the therapeutic effects of such PSCI treatments and their mechanisms of action remain unclear, this study evaluated the effect of 8-week exposure to low-frequency rTMS, 5 days per week, on the cognitive function of patients with PSCI. The therapeutic effect of the treatment was evaluated *via* a cognitive assessment scale and neurophysiological P300 index. Moreover, this study investigated its possible mechanisms of action, providing a basis for the clinical use of low-frequency rTMS therapies.

## Materials and methods

### Participants

A total of 36 patients with PSCI aged 38–75 years were selected from both Xuzhou Rehabilitation Hospital and the Rehabilitation Department of Xuzhou Central Hospital from September 2018 to June 2020. The inclusion criteria were as follows: a PSCI diagnosis as per the expert consensus on the management of post-stroke cognitive impairment of 2017 (1); a previous history of stroke that was confirmed by either brain CT or MRI examinations; cognitive impairment of MMSE grade < 24 (junior high school and above)/20 (primary school)/17 (illiteracy); a stroke within the past 6 months; a signed informed consent form, which had been approved by the ethics committee of Xuzhou Central Hospital, by either the participant or their guardian. The exclusion criteria were as follows: occurrence of other cerebral diseases such as traumatic brain injury, intracranial tumors and infections, and hypoxic-ischemic encephalopathy; occurrence of other conditions that affect cognitive function, such as mental illness, high alcohol consumption, and drug abuse; severe complications that possibly result in patients with unstable conditions; serious hearing or visual impairments; the presence of foreign metal objects either in the cranium such as aneurysm clips, or the body such as pacemakers; a history of epilepsy and malignant tumors; pregnant women and other patients who were unsuitable for the planned treatments.

### Study intervention

#### Group allocation

**Figure 1:**
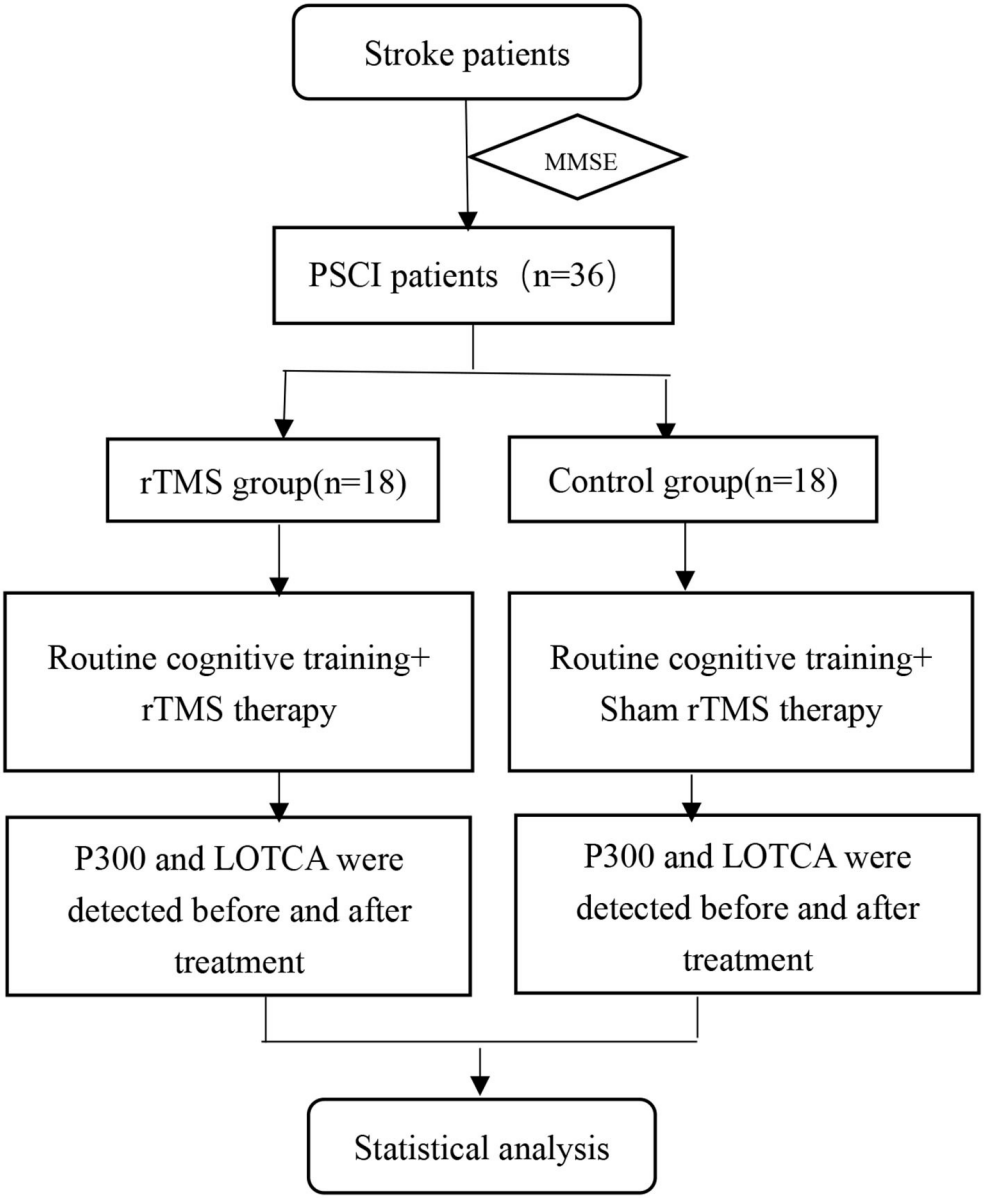
Schematic representation of the treatment design.

The patients were divided into either LF-rTMS treatment (*n* = 18) or control (*n* = 18) groups using the random number table method. The baseline data, loewenstein occupational therapy cognitive assessment (LOTCA) scales, and P300 waves for patients were obtained prior to the first treatment. In terms of treatments, all patients received routine cognitive training, whereas patients in the LF-rTMS group additionally received 1Hz rTMS stimulations. After 8 weeks, all patients received a second assessment of LOTCA scales and their P300 waves (Figure 1).

The rTMS treatment group had 13 men and 5 women. Its average age, course of the disease, and duration of formal education were 60.39 ± 10.87 years, 58.11 ± 28.89 days, and 8.67 ± 3.55 years, respectively. All members in this group were right-handed, there were 10 and eight cases of cerebral infarction and hemorrhage, and there were 17 and one cases with lesion of the left hemisphere and right hemisphere, respectively. The control group had 12 men and six women. Its average age, course of the disease, and duration of formal education were 59.50 ± 11.25 years, 58.39 ± 24.70 days, and 8.50 ± 3.45 years, respectively. All members in this group were right-handed, there were 11 and seven cases of cerebral infarction and hemorrhage, and there were 16 and two cases with lesion of the left hemisphere and right hemisphere, respectively.

The two groups did not significantly differ in gender, age, duration of formal education, course of the disease, type of stroke, lateralization of the lesion, and other general conditions (*P* > 0.05; Table 1).

**Table 1:** Comparison of baseline data of two groups.

**N**		**Gender (n)**		**Age (years, $\bar{x}$ ±s)**	**Duration (days, $\bar{x}$ ±s)**	**Education (years, $\bar{x}$ ±s)**	**Types(n)**		**Lateralization of the lesion**	
		**M**	**F**				**Cerebral infarction**	**Cerebral hemorrhage**	**Left(n)**	**Right(n)**
rTMS	18	13	5	60.39 ± 10.87	58.11 ± 28.89	8.67 ± 3.55	10	8	17	1
Control	18	12	6	59.50 ± 11.25	58.39 ± 24.70	8.50 ± 3.45	11	7	16	2

There was no significant difference between the two groups (P > 0.05).

### Routine cognitive functional training

The appropriate drugs were administered in both groups to control chronic conditions, such as hypertension and diabetes. All patients were also treated with routine cognitive functional training. For PSCI patients, cognitive training plans were designed as per their evaluation results and then delivered as personalized cognitive training as follows.

#### Training for orientational dysfunction

For patients with difficulties in movement due to directional inabilities, they were trained, through repeated practices, to remember their right hands and that on the outside of their right hand is the right side. Moreover, they were similarly trained to remember the common lines from the ward to the physical therapy room.

#### Training for perceptual impairment

##### Training for unilateral space neglect

For this, therapists stood on the neglected side of the patients while talking with them and providing various sensory stimuli to their neglected limbs, such as touch, percussion, massages, and cold objects. Patients were trained to cross the midline with their other hand to retrieve objects they needed, which were on their neglected side. The therapist then randomly drew 40 line segments and a red line on a sheet of A4 paper, ignoring the lateral apex, and encouraged the patient to follow the outline from the apex. This was repeatedly practiced.

##### Training for agnosia

Patients were instructed to remember the names of important relatives by repeatedly looking at photographs. Moreover, patients with visual agnosia were repeatedly trained to not only discriminate items, shapes, and colors but also make full use of other sensory stimuli, such as touch and sound. This training was repeatedly practiced. The variety of colors gradually increased as the patient's cognitive ability improved.

#### Training for attentional impairment

##### Guess assignment

Two transparent cups and a pinball were selected, and while the patient watched, one glass was placed upside down over the pinball. The patient was then requested to point out which glass had the pinball. This was practiced repeatedly. After correct guesses, the training was done with opaque rather than transparent cups. After correct guesses, the training was expanded to use more opaque cups. This was repeatedly practiced.

##### Delete job

Several capitalized Chinese Pinyin letters such as K, B, F, H, G, B, S, G, V, B, M, and l were written in the middle of a paper. The patient was then instructed to delete the letters specified by the therapist, such as “B.” After correct deletions, the order of letters was changed. This was practiced repeatedly.

#### Training for disturbance of thought

##### Extraction of information

The patient was asked to find different kinds of information, such as advertisements and news, from the day's newspaper.

##### Sorting order

The patient was asked to arrange numbers according to value, month, and year. Relatedly, they were asked to arrange pictures of the story in order and subsequently narrate the plot. When successful, the number of pictures gradually increased.

##### Item classification

Patients were given cards that had items in five categories—food, furniture, clothing, household appliances, and grooming products—and each category had five items. Patients were then asked to classify the items and state the reasons behind their classifications. This was practiced repeatedly.

##### Problem-solving ability

Patients were requested to solve hypothetical problems of different levels of complexity, such as what to do if they forgot their keys when going out or what to do if they got lost. The patient was requested to solve other hypothetical problems related to daily life, such as cake sharing and making a schedule.

##### Calculation training

By using either digital games or homework to train computing power, calculations—with content related to daily life—such as shopping in the supermarket, were designed and given to the patient to solve.

##### Memory training

Using the picture memory method, the patients were given a certain number of pictures and then requested to name them before they were repossessed. After a few minutes, the patients were requested to recall the pictures. For memory training on daily activities, a routine for regular daily activities for a patient was established, and the patient was requested to keep to it. Therapists actively participated in completing this training through the use of visual, audio, tactile, and olfactory sensory inputs.

These 30 min pieces of training were done daily, five times a week, for 8 weeks. The difficulty of each training increased as per the patient's ability.

#### RTMS procedures

Based on the cognitive functional training, the rTMS group was treated with low-frequency repetitive transcranial magnetic stimulations. This was conducted with a rapid2 transcranial magnetic stimulator (The Magstim Company, UK) and a standard figure-eight coil. The coil was positioned over the dorsolateral prefrontal cortex (DLPFC) of the unaffected hemisphere—it was positioned in the F3 or F4 region using the international EEG 10–20 system. The active stimulation session was set to a frequency of 1 Hz and 80% of the individual MT with a total of 30 sequences, each of 20 pulses. For the control group, the same stimulation parameters were used, except that the coil was perpendicular to the patient's skull surface resulting in no magnetic stimulation of the brain. All patients received this treatment daily, 5 days a week for 8 weeks.

#### Cognitive assessment

##### The simplified Chinese version of the LOTCA

This scale was selected as it is simple, comprehensive, and easy to use. It includes six assessment areas (orientation, visual perception, spatial perception, action application, visual movement organization, and thinking operation), with a total of 26 test items and a maximum score of 115. The better the cognitive function, the higher the score. The scale is reliable and was validated for guiding treatments for impaired cognitive functions (7).

##### Detection of P300 waves

Electromyographs (EMGs) were recorded for all patients using an evoked potential instrument (Shanghai Nuocheng, China). The electrodes were placed according to the international electroencephalogram 10–20 system. The recording electrodes were placed at the FZ point, whereas the reference electrodes were placed either at the A1 or A2 points, and the ground wire was placed on the forehead. The auditory oddball sequence was composed of target and non-target stimuli. The frequency of the target stimulus was 2000 Hz, with a 15% probability of occurrence. The frequency of the non-target stimulus was 500 Hz, and the probability of occurrence was 85%. The impedance between the electrode and skin was less than 5 KΩ. The sound intensity was 100 dB, and the target signal was superimposed 50 times. Detected P300 waves were recorded.

LOTCA scale and P300 wave assessments of all patients were performed before treatments and also after 8 weeks of treatment by the same physiatrist to minimize error. The physiatrists who performed the assessments were not only excluded from the rehabilitation treatment but also blinded from the grouping of patients.

#### Statistical analysis

All statistical analyses were done in SPSS version 16.0 statistical software. For count data, chi-square tests were used to determine the statistical significance of differences between expected and observed results. Measurement data were expressed as mean ± standard deviation ($\bar{x}$ ± s) and that of both groups were first tested for normality and homogeneity of variance. Paired *t*-tests were used to assess the statistical significance of differences due to treatment within the group, and *t*-tests were used to compare the statistical significance of differences between the groups. An alpha level of 0.05 was used.

## Results

### Effect of cognition therapies

**Figure 2:**
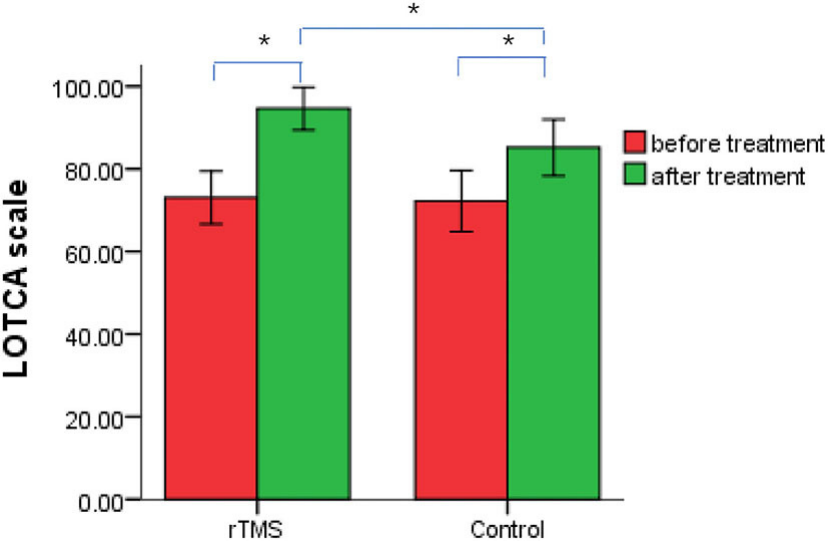
Changes in loewenstein occupational therapy cognitive assessment scores in treatment and control groups of post-stroke cognitive impairment patients, before and after repetitive transcranial magnetic stimulation therapy. *P < 0.05.

Before treatment, there were no significant differences in LOTCA scores between the two groups (*P* > 0.05). After treatment, the LOTCA scores of both groups were significantly higher than those before treatment (*P* < 0.05), and those of the rTMS treatment group were significantly higher than those of the control group (*P* < 0.05; Figure 2).

### Effects of RTMS therapy on P300 waves

**Figure 3:**
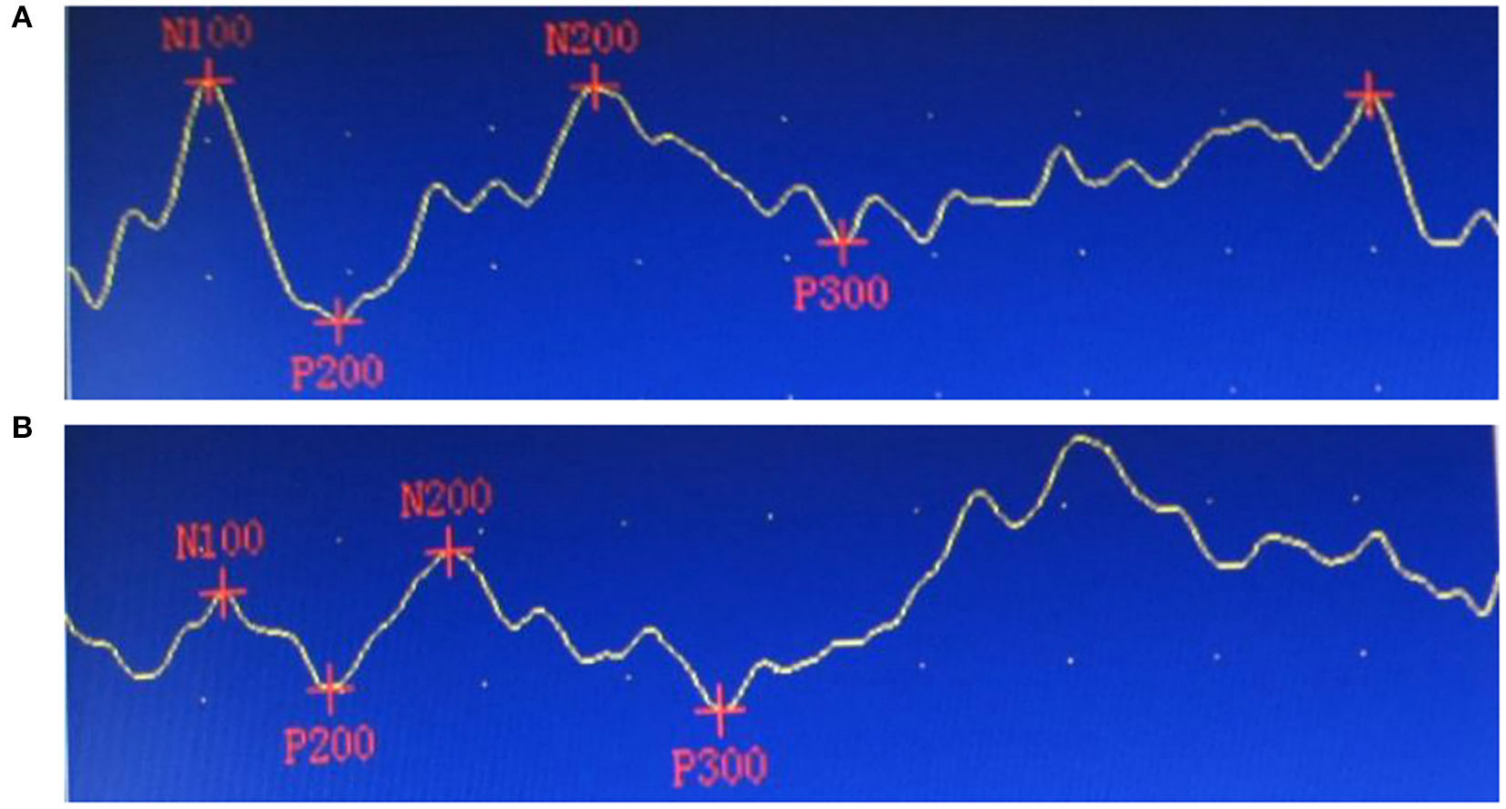
Event-related potential P300 waves of a 56-year-old patient, before and after repetitive transcranial magnetic stimulation treatment. **(A)** Before treatment, P300 latency 403.50 ms, P300 amplitude 5.97 μV, Loewenstein Occupational Therapy Cognitive Assessment (LOTCA) total score, 75 points. **(B)** After treatment, P300 latency 346.50 ms, P300 amplitude 11.30 μV, LOTCA total score, 102 points.

**Figure 4:**
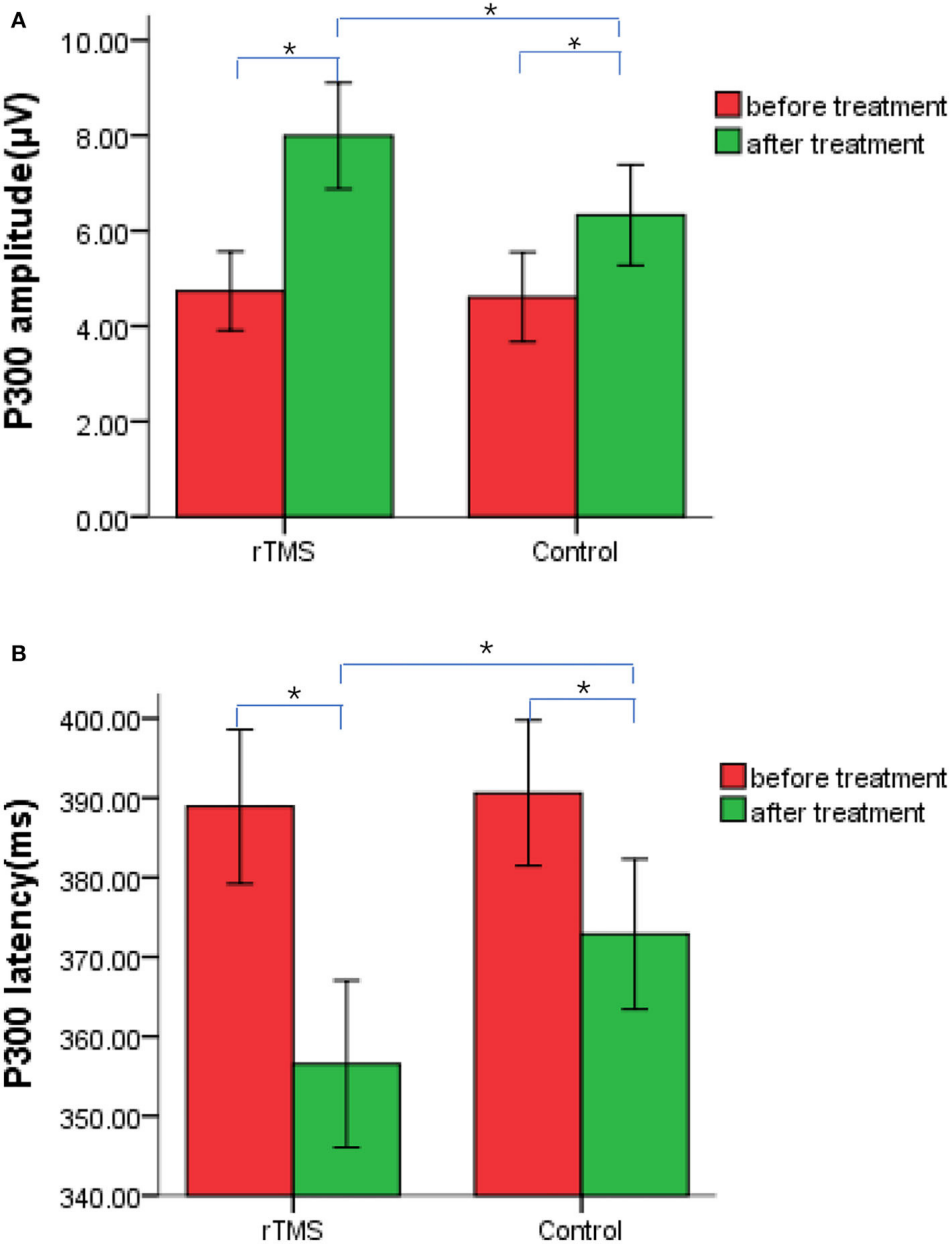
Differences in p300 wave latencies and amplitudes before and after treatment with repetitive transcranial magnetic stimulation (rTMS) therapy in patients from both the rTMS treatment and control groups. **(A)** P300 amplitude. **(B)** P300 latency. *P < 0.05.

Before treatment, P300 wave latencies and amplitudes did not significantly differ between groups (*P* > 0.05). After treatment, P300 latencies and amplitudes were shorter and greater than before treatment (*P* < 0.05), and those of the rTMS group were significantly shorter and greater than those of the control (*P* < 0.05; Figures 3, 4).

## Discussion

RTMS is a non-invasive brain stimulation technique where short, high-intensity magnetic field pulses and changes in the cortical nerve electrical activity are applied to the central nervous system to stimulate nerve cells to produce physiological changes. Historically, rTMS was used to study the potential areas of cognitive processes in the brain (8). Today, the therapeutic effect of rTMS on cognitive impairment is hypothesized to be related to factors, such as stimulation site and rTMS parameters (9). Therefore, the choice of appropriate treatment mode and parameters is crucial for successful therapeutic applications of rTMS. After a stroke, there exists imbalanced excitability between the cerebral hemispheres (10). The excitability of the contralateral brain increases, whereas that of the cortex in the affected side decreases, which causes the cortex of the contralateral side to excessively suppress the affected side, resulting in post-stroke dysfunctions. Varying (11) rTMS frequencies result in different regulatory effects on the excitability of the cerebral cortex. Low-frequency rTMS (stimulation frequency ≤ 1 Hz) inhibits cortical function and reduces the excitability of the cerebral cortex. On the contrary, high-frequency rTMS (stimulation frequency > 1 Hz) excites cortical function. Currently, DLPFC is the main stimulation target of rTMS for the treatment of neuropsychiatric diseases (12). It is likely that the stimulation of DLPFC restored the rich cholinergic innervation from the basal forebrain that is typically impaired in MCI patients (13) and is important for memory tasks in healthy people (14). Presently, the consistent view is that DLPFC is the most important node in brain networks, such as working memory and executive control (15, 16). The excitation of this site may promote recovery of the patient's cognitive function by regulating the function of this brain network. High-frequency rTMS stimulation of the left DLPFC improved post-stroke cognitive functions (17, 18), and this is possibly related to the shortened activation duration and increased excitability of the cerebral cortex. However, high-frequency stimulation of the affected side of the brain may not only increase the risk of epilepsy but also lack a good effect on patients with a right side injury. The use of low-frequency rTMS to stimulate the contralateral brain for treating the patient with PSCI is under-investigated. Thus, we investigated the therapeutic effect of low-frequency rTMS for stimulation of DLPFC in the contralateral brain for improvement of cognitive function in stroke patients. Previously, we observed that low-frequency rTMS had a therapeutic effect on cognitive dysfunction after stroke *via* an unclear mechanism. Here, by applying principles of rTMS technology, we further investigated this effect on brain neural electrical activity through nerve potential P300 detection and evaluated its curative effect.

Information processing in human cognitive activities involves the dynamic interaction of multiple neural networks. Brain networks usually coordinate the processing of relevant information. Improper information processing in stroke patients is the basis of most cognitive impairments. Event-related potential P300, also known as “cognitive potential,” is the most commonly used neurophysiological method for detecting cognitive function and reflecting the process of cognitive information processing. It mainly records the brain potential on the surface of the head through average superposition in cognitive processing. P300 latency reflects the speed of external stimulus processing, whereas P300 amplitude reflects the degree of effective resource mobilization during cognitive processing (19). P300 is an objective and sensitive electrophysiological index that can reflect changes in cognitive function (20). Moreover, patients with cognitive impairment after a stroke had P300 waves of prolonged latencies and decreased amplitudes (21). Relatedly, we confirmed that P300 effectively assessed both the presence of and changes in cognitive impairments in stroke patients.

As a therapy, rTMS was used in this study based on conventional rehabilitation treatments. LOTCA scores for both groups improved after treatment, and those of the treatment group were better than those for the control. Low-frequency rTMS improved the cognitive function of patients with PSCI, and the therapeutic effect of low-frequency rTMS combined with conventional cognitive rehabilitation training was better than that of conventional cognitive rehabilitation training only. Moreover, after treatment, the P300 latencies and amplitudes were, respectively, shorter and greater than before treatment—this change was significantly greater in the treatment than in the control group. This demonstrated that low-frequency rTMS improved the cognitive function of patients with PSCI from the perspective of neurophysiology.

In line with the principles of rTMS and the changes in cognitive-related potential P300, we hypothesize that low-frequency rTMS regulates the excitability of the cerebral cortex and stimulates more brain cells to participate in cognitive information processing. These changes may improve the cerebral cortex by promoting long-term potentiation (LTP)—like changes, and this may be one of the main mechanisms by which rTMS aids the treatment of PSCI. It is well known that cognitive function is closely related to the plasticity of synapses in the central nervous system. LTP of the central nervous synapse is a key manifestation of synaptic functional plasticity that reflects the memory process at the synapse level and is the main index for measuring synaptic functional plasticity in brain science research (22). A study (23) has demonstrated that patients develop cognitive impairment after LTP-like cortical plasticity damage. Another study (24) reported that the excitability of brain injury areas and their surrounding areas continued to decrease after stroke, and the induction amplitude of LTP also decreased significantly. There is evidence that rTMS can improve the LTP effect in patients with cognitive impairment (25), thereby improving cognitive function. However, it is not known whether low-frequency rTMS treatment on the DLPFC of the unaffected hemisphere would produce LTP in PSCI patients. It may achieve this biological effect by regulating the functional status of brain networks related to cognitive activities. Similarly, another study reported that repetitive transcranial magnetic stimulation does not only cause long-term enhancement in local neurons but also activates the neural network as a whole (26). The cortical hippocampal circuit is the brain area closely related to learning and memory. It has been found (27) that rTMS may not directly stimulate the hippocampus but indirectly promote the LTP of hippocampal synapses by triggering the release of brain-derived neural growth factor (BDNF), which is crucial for LTP induction related to synaptic plasticity and is closely related to learning and memory processes (28). This role suggests that long-term rTMS treatment may enhance LTP by promoting the release of neurotransmitters and regulating the functional status of interneurons in neural circuits, thereby changing the neural excitability of associated regions in the brain network and reducing the LTP induction threshold. These findings provide a theoretical basis for low-frequency rTMS treatment to promote LTP changes in synaptic plasticity. However, there are also other brain network circuits closely related to cognitive activities. For instance, Alzheimer's disease (29) destroys the integrity of the frontal–parietal network, resulting in severe impairment of cognitive function. Whether low-frequency rTMS treatment in the DLPFC region of stroke patients will have an LTP effect on other cognitive circuits such as frontal–parietal network needs further research.

The other mechanism for this therapeutic effect possibly involves inhibition of the excitability of the contralateral cortex by low-frequency rTMS, thus reducing the inhibitory effect of the contralateral hemisphere on the excitability of the affected hemisphere, thereby regulating the balance of mutual inhibition between cerebral hemispheres. Moreover, the change in excitability is not limited to the stimulation site but can also be transmitted to other related yet remote functional areas *via* complex brain neurophysiological mechanisms (30), hence regulating functional links of cognitive-related neural circuits and actualizing the reorganization of cognitive function (31, 32). Ultimately, this improves cognitive function. In view of the complexity of cognitive activities and brain network connections, neurophysiological methods such as TMS or TMS-EEG could be used to study some neurophysiological properties of the brain such as spike-time-dependent plasticity (33) to further study the impact of low-frequency rTMS on the cortical plasticity of different cognitive brain networks. In addition, neurophysiological methods could be applied to observe the effects of different rTMS stimulation sites (e.g., precuneus and parietal lobe) on cognitive function.

## Conclusion

Low-frequency rTMS improved cognitive function when based on conventional cognitive rehabilitation therapy. It had a defined curative effect and was well tolerated by patients, making it feasible for clinical application. Moreover, no adverse reactions occurred during low-frequency rTMS treatments indicating that the technology is safe and reliable.

### Limitations of the study

The sample size was small, which impeded observations of the impact of rTMS on individual cognitive function. Functional imaging was not used to further investigate the possible therapeutic mechanisms of action.

## Data availability statement

The original contributions presented in the study are included in the article/supplementary material, further inquiries can be directed to the corresponding author.

## Ethics statement

The studies involving human participants were reviewed and approved by the Ethics Committee of Xuzhou Central Hospital. The patients/participants provided their written informed consent to participate in this study.

## Author contributions

BY collected the data and drafted the manuscript. GZ developed the rTMS protocol. WS designed the study and revised the manuscript. GZ and CW recruited patients. BY and WS analyzed the data. All authors contributed to the article and approved the submitted version.

## Funding

This study was supported by the following foundations: Key Science and Technology Planning Foundation of Xuzhou (Grant number: KC19156), Key Health and Health Committee Foundation of Jiangsu Province (Grant number: K2019012), and Clinical Technical Backbone Foundation of Xuzhou (Grant number: 2018gg035).

## Conflict of interest

The authors declare that the research was conducted in the absence of any commercial or financial relationships that could be construed as a potential conflict of interest.

## Publisher's note

All claims expressed in this article are solely those of the authors and do not necessarily represent those of their affiliated organizations, or those of the publisher, the editors and the reviewers. Any product that may be evaluated in this article, or claim that may be made by its manufacturer, is not guaranteed or endorsed by the publisher.

## References

[1] Chinese Stroke Society. Expert Committee on management of post stroke cognitive impairment. Expert consensus on management of post stroke cognitive impairment. Chin J stroke. (2017) 12:519–31. 10.3969/j.issn.1673-5765.2017.06.011

[2] Qu Y Zhuo L Li N Hu Y Chen W Zhou Y. Prevalence of post-stroke cognitive impairment in china: a community-based, cross-sectional study. PLoS One. (2015) 10:e0122864. 10.1371/journal.pone.0122864 25874998 PMC4395303

[3] Barker-Collo S Feigin VL Parag V Lawes CM Senior H. Auckland Stroke Outcomes Study. Part 2: cognition and functional outcomes 5 years poststroke. Neurology. (2010) 75:1608–16. 10.1212/WNL.0b013e3181fb44c8 21041784

[4] Du J Yang F Hu J Hu J Xu Q Cong N. Effects of high- and low-frequency repetitive transcranial magnetic stimulation on motor recovery in early stroke patients: evidence from a randomized controlled trial with clinical, neurophysiological and functional imaging assessments. Neuroimage Clin. (2019) 21:101620. 10.1016/j.nicl.2018.101620 30527907 PMC6411653

[5] Harvey DY Podell J Turkeltaub PE Faseyitan O Coslett HB Hamilton RH. Functional reorganization of right prefrontal cortex underlies sustained naming improvements in chronic aphasia via repetitive transcranial magnetic stimulation. Cogn Behav Neurol. (2017) 30:133–44. 10.1097/WNN.0000000000000141 29256908 PMC5797702

[6] Dionísio A Duarte IC Patrício M Castelo-Branco M. Transcranial magnetic stimulation as an intervention tool to recover from language, swallowing and attentional deficits after stroke: a systematic review. Cerebrovasc Dis. (2018) 46:178–85. 10.1159/000494213 30343304

[7] Yan TB Ma C Guo You H. Validity and reliability of Loewenstein cognitive assessment scale (Simplified Chinese version). Chin J Phys Med Rehabil. (2004) 26:81–4.

[8] Beynel L Appelbaum LG Luber B Crowell CA Hilbig SA Lim W. Effects of online repetitive transcranial magnetic stimulation (rTMS) on cognitive processing: a meta-analysis and recommendations for future studies. Neurosci Biobehav Rev. (2019) 107:47–58. 10.1016/j.neubiorev.2019.08.018 31473301 PMC7654714

[9] Kim BR Kim DY Chun MH Yi JH Kwon JS. Effect of repetitive transcranial magnetic stimulation on cognition and mood in stroke patients: a double-blind, sham-controlled trial. Am J Phys Med Rehabil. (2010) 89:362–8. 10.1097/PHM.0b013e3181d8a5b1 20407301

[10] Miniussi C Rossini PM. Transcranial magnetic stimulation in cognitive rehabilitation. Neuropsychol Rehabil. (2011) 21:579–601. 10.1080/09602011.2011.562689 21462081

[11] Terao Y Ugawa Y. Basic mechanisms of TMS. J Clin Neurophysiol. (2002) 19:322–43. 10.1097/00004691-200208000-00006 12436088

[12] Iimori T Nakajima S Miyazaki T Tarumi R Ogyu K Wada M. Effectiveness of the prefrontal repetitive transcranial magnetic stimulation on cognitive profiles in depression, schizophrenia, and Alzheimer's disease: A systematic review. Prog Neuropsychopharmacol Biol Psychiatry. (2019) 88:31–40. 10.1016/j.pnpbp.2018.06.014 29953934

[13] Xia Y Eeles E Fripp J. Reduced cortical cholinergic innervation measured using [18F]-FEOBV PET imaging correlates with cognitive decline in mild cognitive impairment. Neuroimage Clin. (2022) 34:102992. 10.1016/j.nicl.2022.102992 35344804 PMC8958543

[14] Harel BT Pietrzak RH Snyder PJ. Effect of cholinergic neurotransmission modulation on visual spatial paired associate learning in healthy human adults. Psychopharmacol (Berl). (2013) 228:673–83. 10.1007/s00213-013-3072-2 23568575

[15] Chen AC Oathes DJ Chang C Bradley T Zhou ZW Williams LM. Causal interactions between fronto-parietal central executive and default-mode networks in humans. Proc Natl Acad Sci USA. (2013) 110:19944–9. 10.1073/pnas.1311772110 24248372 PMC3856839

[16] Van den Boom MA Jansma JM Ramsey NF. Rapid acquisition of dynamic control over DLPFC using real-time fMRI feedback. Eur Neuropsychopharmacol. (2018) 28:1194–205. 10.1016/j.euroneuro.2018.08.508 30217551 PMC6420021

[17] Yin M Liu Y Zhang L Zheng H Peng L Ai Y. Effects of rTMS Treatment on Cognitive Impairment and Resting-State Brain Activity in Stroke Patients: A Randomized Clinical Trial. Front Neural Circuits. (2020) 14:563777. 10.3389/fncir.2020.563777 33117131 PMC7561423

[18] Li Y Luo H Yu Q Yin L Li K Li Y. Cerebral functional manipulation of repetitive transcranial magnetic stimulation in cognitive impairment patients after stroke: an fMRI study. Front Neurol. (2020) 11:977. 10.3389/fneur.2020.00977 33013646 PMC7506052

[19] Rossini PM Rossi S Babiloni C Polich J. Clinical neurophysiology of aging brain: from normal aging to neurodegeneration. Prog Neurobiol. (2007) 83:375–400. 10.1016/j.pneurobio.2007.07.010 17870229

[20] Jiang S Qu C Wang F Liu Y Qiao Z Qiu X. Using event-related potential P300 as an electrophysiological marker for differential diagnosis and to predict the progression of mild cognitive impairment: a meta-analysis. Neurol Sci. (2015) 36:1105–12. 10.1007/s10072-015-2099-z 25663086

[21] Suzuki M Hoshiyama M. Difference in P300 response between hemi-field visual stimulation. Neurol Sci. (2011) 32:603–8. 10.1007/s10072-011-0544-1 21468682

[22] Morris RG. Elements of a neurobiological theory of hippocampal function: the role of synaptic plasticity, synaptic tagging and schemas. Eur J Neurosci. (2006) 23:2829–46. 10.1111/j.1460-9568.2006.04888.x 16819972

[23] Di Lorenzo F Motta C Casula EP Bonnì S Assogna M Caltagirone C. LTP-like cortical plasticity predicts conversion to dementia in patients with memory impairment. Brain Stimul. (2020) 13:1175–82. 10.1016/j.brs.2020.05.013 32485235

[24] Di Lazzaro V Pilato F Dileone M Profice P Capone F Ranieri F. Modulating cortical excitability in acute stroke: a repetitive TMS study. Clin Neurophysiol. (2008) 119:715–23. 10.1016/j.clinph.2007.11.049 18165149

[25] Li X Qi G Yu C Lian G Zheng H Wu S. Cortical plasticity is correlated with cognitive improvement in Alzheimer's disease patients after rTMS treatment. Brain Stimul. (2021) 14:503–10. 10.1016/j.brs.2021.01.012 33581283

[26] Pell GS Roth Y Zangen A. Modulation of cortical excitability induced by repetitive transcranial magnetic stimulation: influence of timing and geometrical parameters and underlying mechanisms. Prog Neurobiol. (2011) 93:59–98. 10.1016/j.pneurobio.2010.10.003 21056619

[27] Ogiue-Ikeda M Kawato S Ueno S. The effect of repetitive transcranial magnetic stimulation on long-term potentiation in rat hippocampus depends on stimulus intensity. Brain Res. (2003) 993:222–6. 10.1016/j.brainres.2003.09.009 14642850

[28] Adachi N Numakawa T Richards M Nakajima S Kunugi H. New insight in expression, transport, and secretion of brain-derived neurotrophic factor: Implications in brain-related diseases. World J Biol Chem. (2014) 5:409–28. 10.4331/wjbc.v5.i4.409 25426265 PMC4243146

[29] Di Lorenzo F Ponzo V Motta C Bonnì S Picazio S Caltagirone C. Impaired spike timing dependent cortico-cortical plasticity in Alzheimer's disease patients. J Alzheimers Dis. (2018) 66:983–91. 10.3233/JAD-180503 30372679

[30] Rossi S Hallett M Rossini PM Pascual-Leone A. Safety of TMS Consensus Group. Safety, ethical considerations, and application guidelines for the use of transcranial magnetic stimulation in clinical practice and research. Clin Neurophysiol. (2009) 120:2008–39. 10.1016/j.clinph.2009.08.016 19833552 PMC3260536

[31] Hamilton RH Sanders L Benson J Faseyitan O Norise C Naeser M. Stimulating conversation: enhancement of elicited propositional speech in a patient with chronic non-fluent aphasia following transcranial magnetic stimulation. Brain Lang. (2010) 113:45–50. 10.1016/j.bandl.2010.01.001 20159655 PMC2909623

[32] Barwood CH Murdoch BE Whelan BM Lloyd D Riek S O'Sullivan JD. Modulation of N400 in chronic non-fluent aphasia using low frequency Repetitive Transcranial Magnetic Stimulation (rTMS). Brain Lang. (2011) 116:125–35. 10.1016/j.bandl.2010.07.004 20678791

[33] Markram H Gerstner W Sjöström PJ. Spike-timing-dependent plasticity: a comprehensive overview. Front Synaptic Neurosci. (2012) 4:2. 10.3389/fnsyn.2012.00002 22807913 PMC3395004

